# Correction: Structure-based design, synthesis, and biological activity evaluation of chalcone–piperazine derivatives as dual AChE and MAO B inhibitors

**DOI:** 10.1039/d5ra90135a

**Published:** 2025-11-19

**Authors:** Berkant Kurban, Begüm Nurpelin Sağlık Özkan, Derya Osmaniye, Serkan Levent, Yusuf Özkay, Zafer Asım Kaplancıklı

**Affiliations:** a Department of Pharmaceutical Chemistry, Faculty of Pharmacy, Afyonkarahisar Health Sciences University 03030 Afyonkarahisar Turkey; b Department of Pharmaceutical Chemistry, Faculty of Pharmacy, Anadolu University 26470 Eskişehir Turkey zakaplan@anadolu.edu.tr; c The Institute of Graduate Education, Anadolu University 26470 Eskişehir Turkey; d Central Research Laboratory (MERLAB), Faculty of Pharmacy, Anadolu University 26470 Eskişehir Turkey; e Department of Analytical Chemistry, Faculty of Pharmacy, Anadolu University 26470 Eskişehir Turkey; f Pharmacy Services, Vocational School of Health Services, Bilecik Şeyh Edebali University 11000 Bilecik Turkey

## Abstract

Correction for ‘Structure-based design, synthesis, and biological activity evaluation of chalcone–piperazine derivatives as dual AChE and MAO B inhibitors’ by Berkant Kurban *et al.*, *RSC Adv.*, 2025, **15**, 40897–40911, https://doi.org/10.1039/D5RA05397H.

The authors regret that details of the funding information were omitted from the Acknowledgements of the original manuscript. The correct Acknowledgements are as shown below.

## Acknowledgements

As the authors of this study, we thank Anadolu University Faculty of Pharmacy Central Research Laboratory (MERLAB), for their support and contributions. This study was supported by Anadolu University under the Scientific Research Projects (BAP) program, Project Code: YTT-2025-2710. Additionally, this study is a master’s thesis. YÖK Thesis number: 806986.

The Royal Society of Chemistry apologises for these errors and any consequent inconvenience to authors and readers.

